# Conformational Selection Underlies Recognition of a Molybdoenzyme by Its Dedicated Chaperone

**DOI:** 10.1371/journal.pone.0049523

**Published:** 2012-11-19

**Authors:** Magali Lorenzi, Léa Sylvi, Guillaume Gerbaud, Elisabetta Mileo, Frédéric Halgand, Anne Walburger, Hervé Vezin, Valérie Belle, Bruno Guigliarelli, Axel Magalon

**Affiliations:** 1 Unité de Bioénergétique et Ingénierie des Protéines (UMR7281), Institut de Microbiologie de la Méditerranée, CNRS & Aix-Marseille Univ, Marseille, France; 2 Laboratoire de Chimie Bactérienne (UMR7283), Institut de Microbiologie de la Méditerranée, CNRS & Aix-Marseille Univ, Marseille, France; 3 Laboratoire de Spectrochimie Infrarouge et Raman (UMR8516), Villeneuve d'Ascq, France; University of Cambridge, United Kingdom

## Abstract

Molecular recognition is central to all biological processes. Understanding the key role played by dedicated chaperones in metalloprotein folding and assembly requires the knowledge of their conformational ensembles. In this study, the NarJ chaperone dedicated to the assembly of the membrane-bound respiratory nitrate reductase complex NarGHI, a molybdenum-iron containing metalloprotein, was taken as a model of dedicated chaperone. The combination of two techniques *ie* site-directed spin labeling followed by EPR spectroscopy and ion mobility mass spectrometry, was used to get information about the structure and conformational dynamics of the NarJ chaperone upon binding the N-terminus of the NarG metalloprotein partner. By the study of singly spin-labeled proteins, the E119 residue present in a conserved elongated hydrophobic groove of NarJ was shown to be part of the interaction site. Moreover, doubly spin-labeled proteins studied by pulsed double electron-electron resonance (DEER) spectroscopy revealed a large and composite distribution of inter-label distances that evolves into a single preexisting one upon complex formation. Additionally, ion mobility mass spectrometry experiments fully support these findings by revealing the existence of several conformers in equilibrium through the distinction of different drift time curves and the selection of one of them upon complex formation. Taken together our work provides a detailed view of the structural flexibility of a dedicated chaperone and suggests that the exquisite recognition and binding of the N-terminus of the metalloprotein is governed by a conformational selection mechanism.

## Introduction

Highly specific and tightly regulated interactions between proteins are essential for any life process. Moreover, proteins are inherently dynamic and often sample a vast ensemble of conformations. It is now widely accepted that the dynamic nature of proteins plays a critical role not only in molecular recognition but also in the evolution of molecular interactions [1], [2], [3].

One prominent example is molecular chaperones which adopt numerous structurally distinct conformations to recognize and fold a broad number of different substrate proteins [4], [5], [6], [7]. On the opposite, folding and assembly of metal-containing proteins is ensured by chaperones specific of a given substrate [8], [9]. These dedicated chaperones literally orchestrate several events ranging from metal centers insertion, folding, membrane targeting and even translocation of their substrates. Current models for the mechanism of coordinated assembly of metalloproteins suggest that dedicated chaperones initially recognize and bind to the N-terminus of unfolded metalloproteins, which then recruit components of metal cofactor biosynthesis machineries to form the mature and active metalloproteins [10], [11], [12], [13]. One of the most prominent example is the NarJ chaperone ensuring folding and assembly of the membrane-bound respiratory nitrate reductase complex, NarGHI [9], [14], member of a large group of molybdenum containing enzymes [9], [15]. NarJ coordinates several maturation events through binding to two distinct sites of the NarG catalytic subunit [12]. Interaction with the N-terminus hampers membrane anchoring of an immature complex while interaction at a second site controls sequential insertion of both an iron-sulfur cluster (FS0) and of the molybdenum cofactor [16], [17]. Accordingly, absence of NarJ is associated with a premature membrane anchoring of an immature and inactive NarGH complex to NarI [12], [17]. The NarJ binding epitope on the N-terminus of NarG is restricted to the first 15 residues without any influence on the binding properties [18]. Calorimetric experiments demonstrated that this interaction is mostly driven by hydrophobic interactions and strongly modulated by protonation of NarJ [18]. Furthermore, NMR showed that the amphiphilic helix adopted by the N-terminus of NarG within the X-ray structure of the NarGHI complex [19] is conserved in the isolated NarG(1–15) peptide and remains unchanged upon NarJ binding [18].

Despite a low level of sequence identity, resolution by X-ray crystallography of the structure of several dedicated chaperones for molybdenum containing enzymes indicates a conserved all-helical fold [20], [21], [22], [23], [24], [25] and allows the description of a new family of chaperones (Pfam PF02613) to which NarJ belongs. While X-ray crystallography provided invaluable high-resolution structural information on dedicated chaperones, NMR is, in many cases, a better source to get information about dynamics and flexibility. NMR studies conducted on *E. coli* NarJ suggest the existence of one or several flexible regions by the absence of a number of peaks in the ^1^H,^15^N-HSQC spectrum which also precluded complete assignment of the residues. Similar experiments conducted in presence of the NarG(1–15) peptide showed a drastic modification of the HSQC spectrum interpreted as the result of a global conformational change as the result of complex formation [18]. Altogether, these NMR studies did not provide a detailed picture of the conformational dynamics. Moreover, no 3D structure of the complex between the chaperone and the N-terminus of its cognate partner for any dedicated chaperones is available so far.

A better understanding of the initial steps of the assembly process which consist in the exquisite recognition of the metalloprotein partner through its N-terminus by the dedicated chaperone called for the use of alternative strategies. Site-directed spin labeling combined with EPR spectroscopy is a powerful method for monitoring the structure and dynamics of soluble and membrane proteins of arbitrary molecular weight [26]. In site-directed spin labeling, a radical containing nitroxide spin label is site specifically introduced onto cysteine residues introduced *via* site-directed mutagenesis. The resulting EPR spectrum of the generated paramagnetic side chain can be used to identify interaction sites within protein complexes or to detect changes in protein conformations, and the data can be interpreted in terms of relative domain movement, backbone dynamics and folding or unfolding events [26], [27], [28], [29], [30]. Moreover, pulsed EPR techniques, specifically double electron-electron resonance (DEER) experiments, allow the measurement of long-range distances and distance distributions in multi-labeled proteins [31]. Additional information can be gained through the use of ion mobility (IM) experiments coupled to mass spectrometry which allow differentiating molecules according to their charge state (CS), collision cross-section (CCS) and shape with the determination of drift time curves reflecting gas phase conformation of proteins and peptides [32], [33], [34]. By preserving protein-ligand complexes and the conformational ensemble of proteins in the gas phase, IM has emerged as a powerful tool for the study of macromolecular structures [35], [36], [37], [38]. Such device allows inferring insights on ligand-protein and protein-protein interactions, and unveiling conformational changes upon ligand binding [39], [40], [41], [42].

In this study, we aimed to provide new information on protein dynamics of a dedicated chaperone during the binding process to the N-terminus of its metalloprotein partner by the combination of the two above-mentioned complementary biophysical approaches using the NarJ/NarG(1–15) peptide as a working model. Here, we clearly identified the location of the interaction site between the two partners and analyzed in detail the structural flexibility properties exhibited by the dedicated chaperone NarJ. In addition, we demonstrated that partner binding results from the selection of an accessible conformation and its further rearrangement induced upon recognition.

## Materials and Methods

### Protein expression and purification

Mutants were generated by site-directed mutagenesis of the pDSNarJT-6His and mutations were verified by DNA sequencing. Overexpression and purification of NarJ truncated of the last 50 amino acid residues (referred as NarJT) and carrying a C-terminal hexahistidine tag were carried out as described previously [14], [18].

### N-terminal NarG peptide

The NarG(1–15) peptide was chemically synthesized and purified by Synprosis (Marseille, France) as previously reported [18].

### Spin labeling of NarJT

Prior to spin labeling, reduction of cysteine residues was carried out by incubating the NarJT variants in 20 mM of DTT for 30 min at room temperature. DTT was removed by PD10 desalting column (GE Healthcare) using a 50 mM Tris-HCl pH 8.0, 100 mM NaCl buffer. Spin label 1-oxyl-2,2,5,5-tetramethyl-pyrroline-3-methyl methanethiosulfonate, MTSL, (Toronto Research Chemicals Inc.) was immediately added to the sample at a molar excess of 10 for mono or 20 for double labeling. The reaction was carried out during one hour for mono spin labeling and four hours for double spin labeling with four consecutive additions of MTSL (one per hour) in the dark and at 277 K under gentle stirring and a continuous flow of argon. The excess of unbound spin label was removed by PD10 desalting column with the same elution buffer described above.

### Circular dichroïsm

CD spectra were recorded on a Jasco 815 CD spectrometer using 1-mm thick quartz cells in 10 mM sodium phosphate pH 7.5 at 296 K. CD spectra were measured from 260 to 190 nm, at 20 nm/min and were averaged from 2 scans. The spectra were corrected for buffer signal. Mean ellipticity values per residue ([θ]) were calculated as described previously [43]. Protein concentrations of 0.1 mg/mL were used.

### Tryptophan fluorescence spectroscopy

Intrinsic tryptophan fluorescence emission was measured by using a Fluorolog FL3–21 spectrofluorimeter and a 1-cm pathlength cuvette at 296 K. A NarJT concentration of 2 µM in a buffer containing 50 mM Tris-HCl pH 7.5, 1 mM MgCl_2_ was used. Increasing concentrations of NarG(1–15) peptide at 50 µM (initial concentration) were then added and the emission fluorescence was scanned. Excitation was at 285 nm and emission was recorded from 310 to 410 nm with excitation and emission band-passes at 2 nm. Binding of the peptide was monitored by recording the variation of intrinsic tryptophan fluorescence of NarJT produced after addition of increasing concentrations of the peptide. Corrections for both the variation of volume and the inner-filter effect of the peptide were performed under the same conditions by using *N*-acetyl-L-tryptophanamide instead of NarJT. A 30% decrease of the tryptophan fluorescence was maximally observed upon peptide addition. Dissociation constants were calculated by plotting relative fluorescence peak integration against ligand concentration and curve-fitting using Origin software.

### Continuous Wave EPR

Room temperature experiment (296 K) EPR spectra were recorded on an ESP 300E Bruker spectrometer equipped with an ELEXSYS Super High Sensitivity resonator operating at X-band (9.9 GHz). The microwave power was 10 mW, the magnetic field modulation amplitude was optimized to avoid over-modulation of the signal (in the range of 0.1 mT to 0.3 mT) and the frequency modulation was 100 kHz. Spin quantitation was carried out as described previously [44] and gave a labeling yield of ∼80% for mono spin labeling (H21C, Q104C, Q149C, and E119C) and 190% for double spin labeling (H21C/Q104C). Labeled NarJT variants were studied in the absence and presence of the NarG(1–15) peptide in a 10-fold molar excess in a 50 mM Tris-HCl pH 7.9, 100 mM NaCl buffer.

### Simulation of the EPR spectra

The EPR spectra recorded at room temperature were simulated using the EPRSIM-C software program. This program, kindly provided by Dr. J. Strancar (University of Ljubljana, Slovenia), is based on the so-called motional-restricted fast-motion approximation described in details in [45]. Such analysis is well adapted to analyze structural changes in proteins [46]. Briefly, the partial averaging of the hyperfine and *g* tensors is described in this model by the following parameters: an effective rotational correlation time τ and two angles (θ_0_ and φ_0_) corresponding respectively to the amplitude and the anisotropy of the spin label rotational motion within a cone. The normalization of the two last parameters by Ω = (θ_0_φ_0_)/(π/2)^2^ represents the free rotational space, which varies from zero (totally restricted movement) to 1 (totally unrestricted movement). Two other parameters were required for simulation: a residual width (ω) and a scalar parameter linked to the polarity of the probe environment (p_A_) for the adjustment of the hyperfine tensor principal values. Fits were performed by minimizing the χ^2^ value.

### Double electron electron resonance (DEER) experiments and Distance Analysis

NarJT samples with and without NarG(1–15) peptide were prepared in a 50 mM Tris-HCl pH 7.9, 100 mM NaCl buffer. In a second set of experiments, to prevent heterogenous protein concentrations that lead to a shortening of the spin label relaxation time and then to a decrease of the DEER signal to noise ratio, the NarJT samples were prepared in the presence of 30% (v/v) glycerol used as cryoprotectant. As glycerol is known to potentially modify binding affinities and in particular hydrophobic interactions, it has been added only after complex formation and just before rapid freezing in liquid nitrogen. For each sample, the final protein concentration was 115 µM in each case. Experiments were conducted on a Bruker ELEXSYS E580 spectrometer at X-band (9.9 GHz) using the standard MD5 dielectric resonator and at Q-band (34 GHz) using the standard EN 5107D2 resonator. The system was equipped with an Oxford helium temperature regulation unit and the data were acquired at 60 K. This temperature has been optimized according to the relaxation times measured at variable temperatures in the range of 20–100 K with 10 K steps. The four-pulse DEER experiment ((π/2) ν_1_−τ_1_−(π)_ν1_−τ−(π)_ν2_−τ_1_+τ_2_−τ−(π)_υ1_−τ_2_−echo) was used with observe (ν_1_) pulse durations of 16 ns (π/2) and 32 ns (π) at X-band and 20 ns (π/2) and 40 ns (π) at Q-band. For both X-band and Q-band experiments, interpulse delays τ_1_ of 200 ns were used and τ_2_ was adjusted depending on the phase memory constant time, T_m_, of the sample [47]. Initial τ of 100 ns was used with increment of 4ns. The pump ELDOR pulse (ν_2_, 32ns) was centered at the central resonance and the observed frequency was set at ν_1_−ν_2_≈72 MHz away on the low field side. In both X and Q-band DEER experiments, the total acquisition time was between 8–12 hours corresponding to 40–60 averaged scans. Signal processing was achieved using the DeerAnalysis2011 software package under Matlab [48]. Background echo decay was corrected by using a homogeneous three-dimensional spin distribution and second order polynomial baseline correction. Tikhonov regularization was applied to the corrected dipolar evolution dataset to obtain inter-spin distance distributions using L-curves [49], [50]. For each sample, the optimal regularization factor, representing a compromise between smoothness and resolution, was α = 100 according to L curve criterion [49], [50].

### Electrospray ionization mass spectrometry (ESI-MS) and Ion Mobility (IM) experiments under non denaturing conditions

ESI-MS spectra of NarJT protein were recorded under non denaturing conditions in the positive ion mode using an electrospray ion source fitted on a quadrupole-time of flight hybrid mass spectrometer equipped with a traveling wave ion mobility (TWIM) cell (Synapt HDMS G1, Waters Corp., Manchester). Protein solutions were introduced at a 3 µL/min flow rate. For ESI-MS studies, backing pressure, capillary voltage, sampling cone voltage, extracting cone voltage, source and desolvation temperatures were 4 mbar, 3 kV, 130 V, 4 V, 373 K and 423 K, respectively. For IM experiments performed under the TWIM mode general instrumental parameters were kept as above. Specific parameters related to IM segregation of ions were: gas pressure in ion mobility cell = 0.6 mbar; gas flow rate in the trap cell = 4.5 mL/min; wave height and wave velocity of the trap and transfer cells were 0.2 V; 300 m.sec^−1^ and 3 V and 248 m.sec^−1^, respectively. Wave height and wave velocity in the ion mobility cell were 6 V and 150 m.sec^−1^, respectively. Mass spectra were recorded for 2 min and 15 min for ESI-MS and IM experiments, respectively. Calibration was achieved using a 1 mg/ml Cesium Iodide solution dissolved in 70% 2-propanol (Sigma Aldrich). Prior to MS analyses NarJT protein was extensively washed using ammonium acetate solutions from 1 M to 25 mM concentration range and used at a working concentration of 30 µM. MS spectra analysis gave experimental molecular weights of 22000.0±0.5 Da for NarJT and 1951.16 Da±0.25 Da for NarG(1–15) peptide, in agreement with the theoretical values (Table S2). NarJT:NarG(1–15) peptide ratios of 1∶1 and 1∶3 were used. For CCS calculation of NarJT, a myoglobin solution at 10 µM under denaturing condition was used as calibrate. Rigorous identical experimental IM parameters were used to obtain a calibration curve using the Drift Scope CCS calculator™.

## Results

### Generation of surface-exposed cysteine NarJT variants

**Figure 1:**
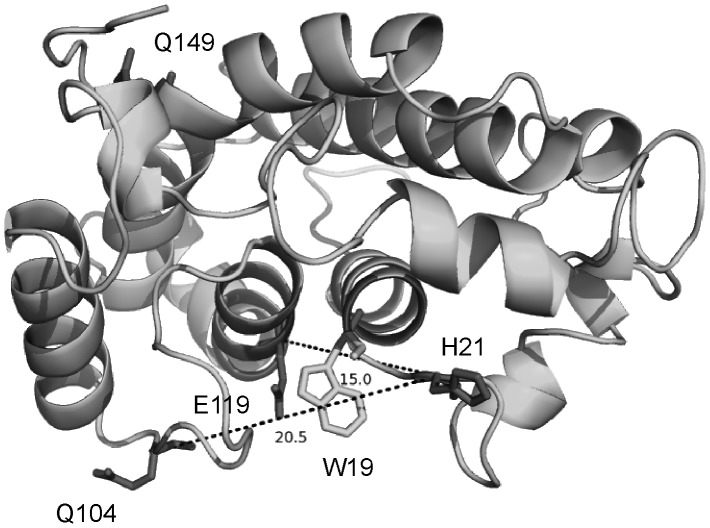
Model structure of NarJT. The positions of the four residues targeted for cysteine mutations and spin labeling are indicated. The position of the single Trp residue (W19) used for binding affinity measurement is indicated as well as the estimated distances between the Cα of H21 and Q104 or H21 and E119. NarJT is represented in cartoon by Pymol (http://www.pymol.org).

A prerequisite for site-directed spin labeling studies is the site-specific introduction of cysteine residues into regions of interest. In absence of structural data for *E. coli* NarJ, positions for the introduction of cysteine residues were selected from the structural model of a truncated form of NarJ named NarJT [18]. This 3D model has been built by homology modeling and was truncated from its 50 C-terminal amino acids because of a lack of similarity. The corresponding NarJT protein was proven to be a valuable construct to study the molecular mechanism of recognition and binding of the N-terminus of the metalloprotein partner as its binding parameters towards the NarG(1–15) peptide remained unchanged as compared with full-length NarJ [18]. Furthermore, docking calculation analysis predicted that the NarG(1–15) peptide interacts within a highly conserved elongated hydrophobic groove of NarJT [18]. With respect to this hydrophobic cavity, four positions were selected for mono-spin labeling: position 119 located inside the cavity, 149 on the opposite side, 21 and 104 on both sides of the cavity (Figure 1). Moreover, a double cysteine variant of NarJT (H21C,Q104C) has been produced.

To determine a possible influence of the mutations and of the presence of the spin label on the structural integrity of NarJT, CD spectra were recorded for all NarJT variants before and after labeling. All CD spectra were similar, showing a shape typical of all-helical proteins with two characteristic minima found at 222 and 208 nm (Figure S1). Hence, neither the substitutions nor the spin label influences the global folding of NarJT (Figure S1). Furthermore NarG(1–15) peptide binding activity of the NarJT variants (before and after spin labeling) was evaluated by intrinsic tryptophan fluorescence quenching thanks to the presence of a unique tryptophan residue (W19) located within the above-mentioned hydrophobic cavity (Figure 1). The data clearly showed a nearly unchanged dissociation constant (Table S1). Altogether, CD and fluorescence data demonstrate that neither mutation nor labeling had an effect on both structural integrity and the ability of NarJT to bind its partner.

### Dynamics of nitroxide side chain grafted on NarJT reveals the interaction site with NarG(1–15) peptide

**Figure 2:**
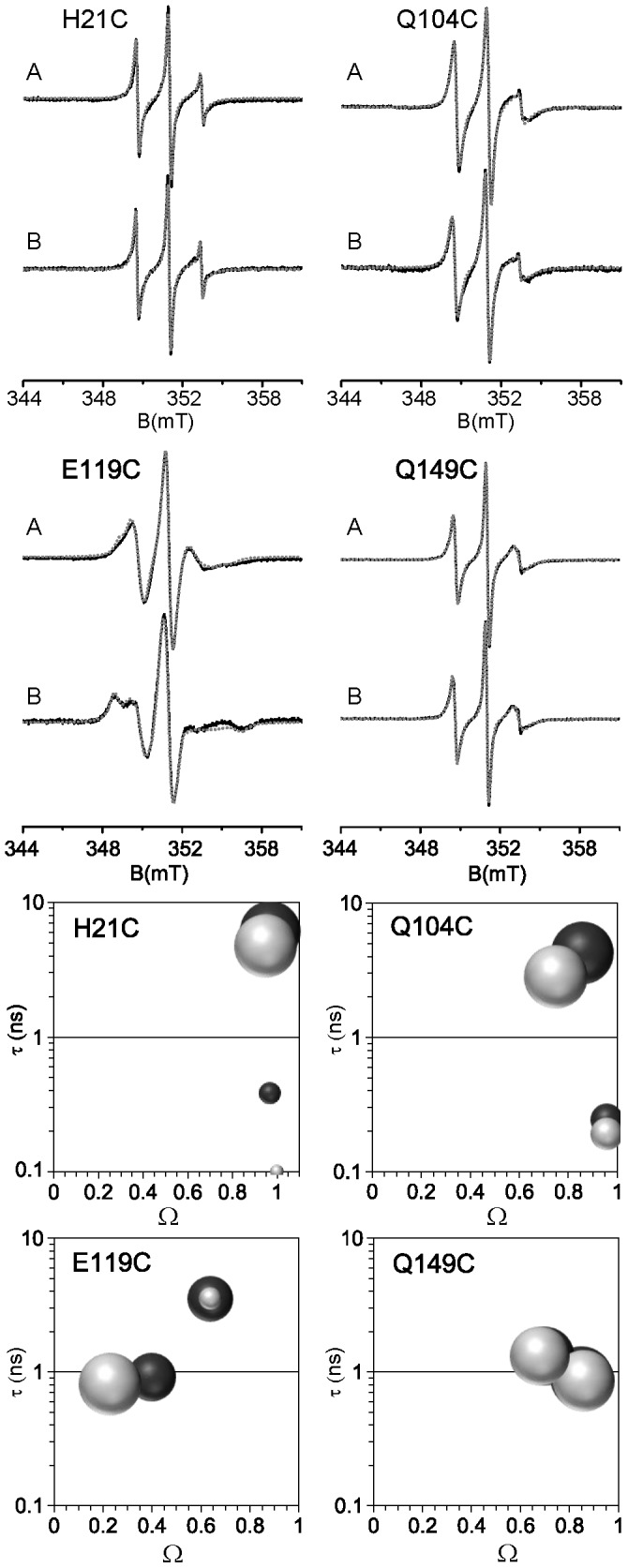
Dynamic studies of the different labeled sites of NarJT. Upper panel: Room temperature EPR spectra of the different labeled NarJT variants in the absence (A) and presence of 10-fold molar excess of the NarG(1–15) peptide (B) in 50 mM Tris-HCl pH 7.9, 100 mM NaCl buffer. Simulated spectra (dotted grey line) are superimposed on the experimental spectra (solid black line). Simulation of the spectra was conducted as described previously [44]. Lower panel: simulated parameters given as [τ-Ω] plots, with τ the effective rotational correlation time in ns and Ω the normalized free rotational space, in the absence (dark gray spheres) and presence (light gray spheres) of the peptide. The area of the spheres is proportional to the relative contribution of the EPR spectral shape components.

Room temperature EPR spectra of nitroxide spin label grafted on proteins are very sensitive to the motion of the label side chain leading to a partial motional averaging of the anisotropic components of the g- and hyperfine tensors [26], [30], [51], [52]. Figure 2 (upper panels) shows room temperature EPR spectra of the four labeled NarJT proteins recorded in the absence and presence of NarG(1–15) peptide. The visual inspection of the different spectra clearly shows a drastic change of the EPR spectrum after peptide binding only for the label at position 119. All EPR spectra were simulated so as to achieve a more detailed description of the impact of the NarG(1–15) peptide on the spin label mobility. The simulation allows decomposition of an EPR spectrum into possibly different components, each of them being described by two main parameters: the effective rotational correlation time τ and the free rotational space Ω (see Materials and Methods). Figure 2 (bottom panels) gives a representation of the results as [τ-Ω] plots for the different positions of the spin label either in the absence or in the presence of NarG(1–15) peptide, with the surface of the spheres being proportional to the relative contribution of the various spectral components (Table 1).

**Table 1:** Simulation parameters of room temperature EPR spectra.

		− NarG(1–15) peptide			+ NarG(1–15) peptide		
Sample	Component	τ (ns)	Ω	%	τ (ns)	Ω	%
H21C	1	6.0±0.6	0.97±0.05	89	4.7±0.6	0.95±0.05	96
	2	0.4±0.1	0.97±0.05	11	0.10±0.05	1.00±0.05	4
Q104C	1	2.8±0.3	0.75±0.05	79	4.4±0.5	0.80±0.05	79
	2	0.24±0.05	0.96±0.05	21	0.26±0.05	0.94±0.05	21
E119C	1	0.9±0.2	0.40±0.05	53	0.8±0.2	0.23±0.05	89
	2	3.5±0.4	0.64±0.05	47	3.5±0.4	0.64±0.05	11
Q149C	1	0.9±0.2	0.85±0.05	53	0.8±0.2	0.86±0.05	52
	2	1.3±0.2	0.70±0.05	47	1.3±0.2	0.68±0.05	48

Parameters (effective rotational correlation time τ, free conformational space Ω and proportion in %) extracted from the simulation of the RT EPR spectra of the labeled NarJT at positions 21, 104, 119 and 149 either in the absence or in the presence of a molar excess of 10 of NarG(1–15). Simulations were performed using the EPRSIM-C software [44].

For positions 21 and 104 located on both sides of the hydrophobic cavity, the corresponding EPR spectra are well-simulated with two components of unequal weights (Figure 2). Upon addition of the NarG(1–15) peptide, no significant EPR spectral change is observed for the main component of the position 21, indicating that the environment of the spin label remains unchanged after peptide binding. For the main component of the spin label grafted at position 104, peptide binding induces a slight spectral change as revealed by the shift of the sphere in the [τ-Ω] plot. This observation indicates that small structural rearrangements occur in the region surrounding the residue 104 upon peptide binding. For these two positions, the minor component reflects a very high mobility of the spin label (Ω close to 1 and low values of τ) that could result from free radicals in solution or a small fraction of labeled unfolded NarJT (Figure 2, Table 1). For positions 119 and 149, the fact that two components of relatively equal weights are required to obtain a good fit indicates that the label experiences different environments (Figure 2 and Table 1). This likely reflects the occurrence of conformational sub-ensembles, possibly corresponding to different orientations of the spin label. Indeed, it has been demonstrated that two components EPR spectra can result from two rotameric states of the nitroxide side chain, each experiencing a unique environment, a behavior frequently found at helical sites [44], [53], [54], [55], [56]. For position 119 located in the hydrophobic cavity, a drastic change in the EPR spectrum is observed consecutive to the addition of NarG(1–15) peptide (Figure 2). These changes are well illustrated in the [τ-Ω] plot where the two components of equal weights in the absence of peptide evolve into one major component (89%) and a minor one (11%). The latter can be attributed to an unbound fraction of NarJT as the Ω and the τ parameters are unchanged as compared to NarJT alone. The major component reflects a highly restricted mobility of the spin label, as indicated by the very low value of Ω, suggesting that the label is in a buried site consecutive to the association of the peptide with NarJT. On the opposite side of the hydrophobic cavity (position 149), no significant variation in the EPR spectra is observed, indicating that the structural characteristics around the probe remain unchanged upon peptide binding. Altogether, these results demonstrate that the region surrounding the residue 119 is part of the interaction site with the N-terminus of the NarG metalloprotein partner.

### Flexibility and conformational dynamics of NarJT upon NarG(1–15) peptide binding as deduced from DEER experiments

**Figure 3:**
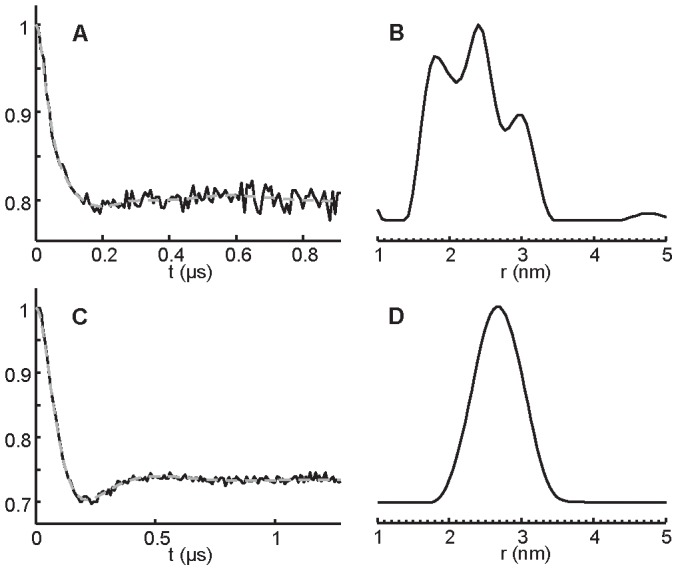
Distance distribution from X-band DEER experiments. (A) and (C): Background corrected DEER time traces (solid line) from a four-pulse DEER experiment on the bi-labeled NarJT (H21C/Q104C) in the absence (A) and presence of 10-fold molar excess of the NarG(1–15) peptide (C) in 50 mM Tris-HCl pH 7.9, 100 mM NaCl buffer. Fits are shown as superimposed gray dotted lines. (B) and (D): Inter-label distance distributions calculated from the DEER time traces by using Tikhonov regularization (DeerAnalysis2011 software package [48]) in the absence (B) and presence of the peptide (D).

Inter-spin distance measurement by DEER techniques is based on the measurement of the dipolar coupling between two interacting spins distant from r, a quantity that is proportional to 1/r^3^. In the DEER sequence [47], the dipolar field modulates the spin-echo envelope and the oscillations of this envelope visualized in the time traces are used to calculate inter-spin distances. Typically, this technique is efficient to measure, under low temperature conditions, inter-spin distances in a wide range going from ∼1.8 nm up to 6.0 nm in proteins [31], [44], [57], thus being of interest for the study of conformational transitions captured in the frozen state. Through the use of a double-cysteine mutant (H21C/Q104C) with spin labels encompassing the binding site, DEER experiments conducted at different frequencies allowed to measure inter-spin distance distribution and its variation induced by the NarG(1–15) peptide binding process. Figure 3 shows the background corrected X-band DEER time traces acquired at low temperature from the bilabeled NarJT in the absence (A) and presence (C) of the NarG(1–15) peptide and the resulting distance distribution obtained by using Tikhonov regularization (B and D) [48] (see Materials and Methods). In the absence of peptide, the distance distribution is broad and composite with the distinction of three maxima (Figure 3B). Such behavior was found to be reproducible using different sample preparations (data not shown). Therefore, this observation suggests the existence of multiple conformations of NarJT. Interestingly, in the presence of the peptide a narrower distance distribution is observed with a unique maximum centered at 2.7 nm (Figure 3D).

**Figure 4:**
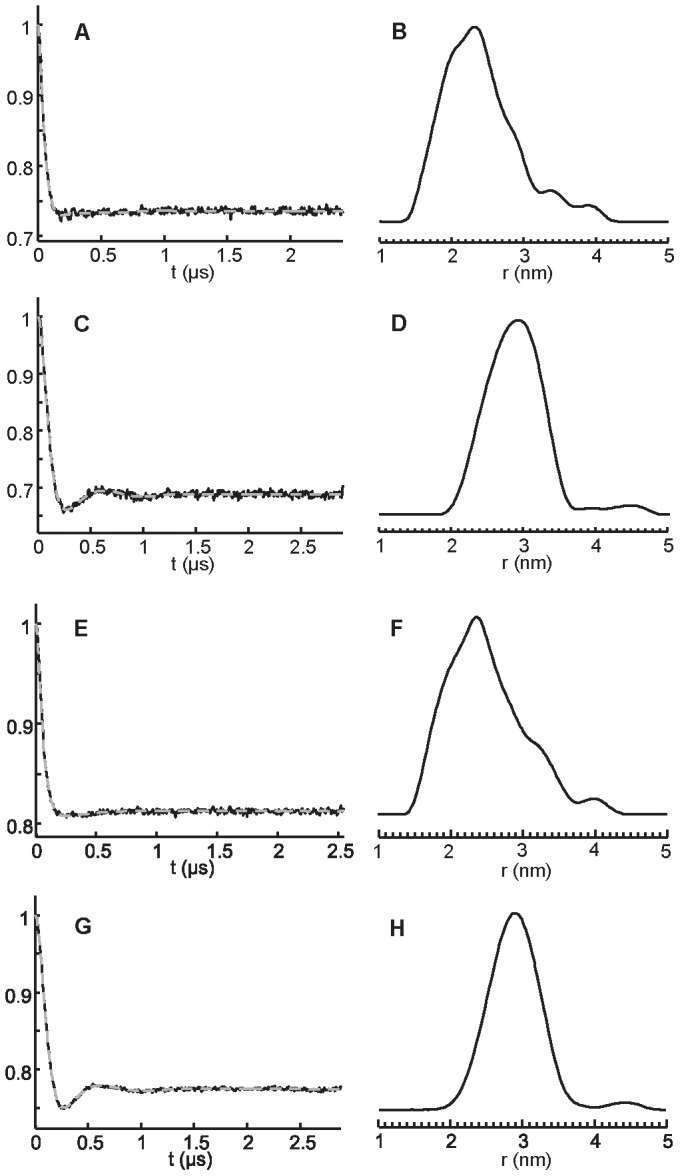
Distance distribution from X-band and Q-band DEER experiments in the presence of 30% glycerol. X-band DEER time traces are shown in (A) to (D) while Q-band DEER time traces are shown in (E) to (G). All samples are prepared in 50 mM Tris-HCl pH 7.9, 100 mM NaCl, 30% (v/v) glycerol buffer. (A) and (C): Background corrected DEER time traces (solid line) from a four-pulse DEER experiment on the bi-labeled NarJT (H21C/Q104C) in the absence (A) and presence of 10-fold molar excess of the NarG(1–15) peptide (C). Fits are shown as superimposed gray dotted lines. (B) and (D): Inter-label distance distributions calculated from the DEER time traces by using Tikhonov regularization (DeerAnalysis2011 software package [48]) in the absence (B) and presence of the peptide (D). (E) and (G): Background corrected DEER time traces (solid line) from a four-pulse DEER experiment on the bi-labeled NarJT (H21C/Q104C) in the absence (E) and presence of 10-fold molar excess of the NarG(1–15) peptide (G). Fits are shown as superimposed gray dotted lines. (F) and (H): Inter-label distance distributions calculated from the DEER time traces by using Tikhonov regularization in the absence (F) and presence of the peptide (H).

Glycerol is often used in DEER experiments prior freezing of the protein sample as a vitrifying agent to prevent excluded volume effects and heterogenous protein concentrations which lead to a drop of the phase memory time (T_m_) and limit the DEER time range [58]. The immediate consequence of the presence of glycerol is generally a longer time for signal evolution leading to a better reliability and resolution of the calculated distance distribution [59], [60]. In this aim, NarJT samples were studied in the presence of glycerol (30% v/v) both in the absence and presence of Nar(1–15) peptide (Figure 4 A,B,C,D). In the absence of the peptide, the different maxima detected previously are less resolved with the observation of shoulders confirming the presence of multiple conformations (Figure 4B). As previously observed, when NarJT is bound to NarG(1–15) peptide, the distribution of distance converges towards a single one centered at 2.9 nm. The difference in inter-spin distance distribution detected without and with glycerol can be attributed to the effect of the cryoprotectant that has been shown to play a role in the compactness of proteins [58], [61].

Finally, DEER experiments were performed at higher frequency (Q-band) for the samples containing glycerol (30% v/v) (Figure 4 E,F,G,H). As demonstrated by Ghimire and coworkers [62], Q-band (34 GHz) increases DEER sensitivity as compared to X-band (9.4 GHz), thus leading to a better signal to noise ratio of the DEER time traces and consequently a more reliable distance distribution. Improved sensitivity can also enable the detection out of the noise of low-frequency components (longer inter-spin distance). In our case, the distance distributions deduced from the Q-band DEER time traces are very similar to those calculated from X-band (Figure 4), confirming that peptide binding induces a transition in distance distribution going from multiple maxima to a single one with a global increase of the distance between the labeled protein regions. This observation highlights the structural dynamics of NarJT during the initial steps of assembly of the metalloprotein partner.

### ESI-MS and ESI-TWIM-MS experiments substantiate a conformational selection mechanism for NarG(1–15) peptide binding

**Figure 5:**
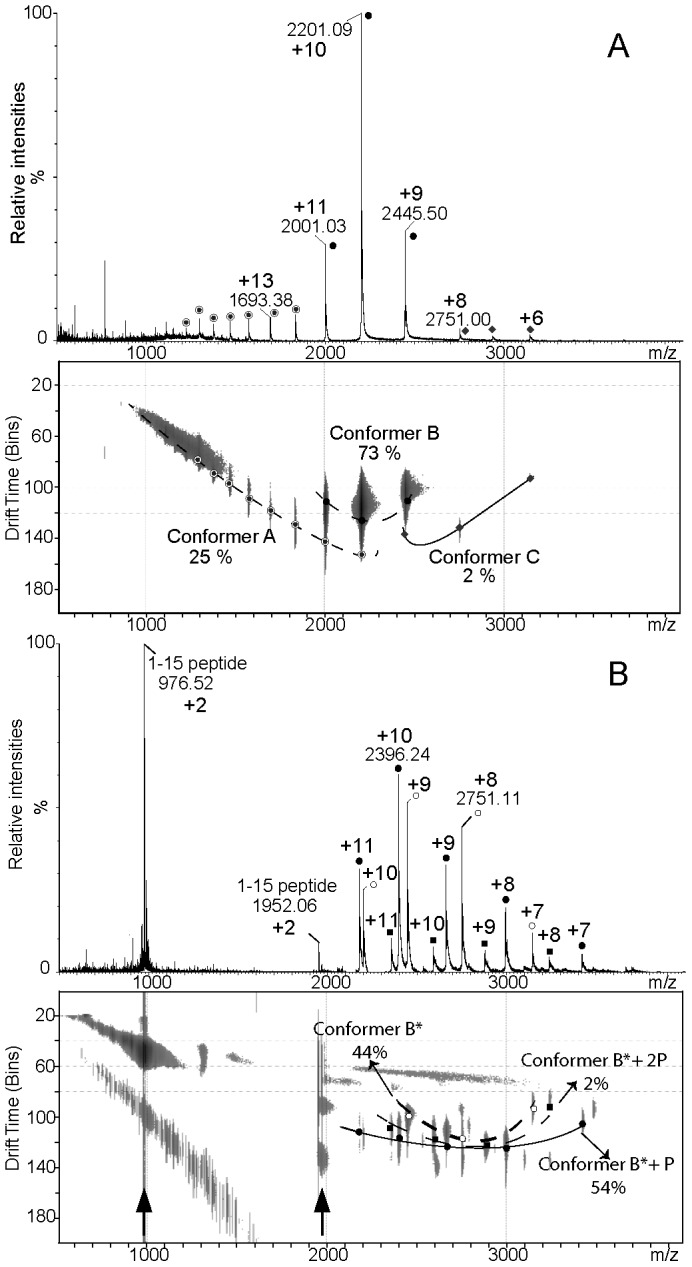
Mass spectra and drift charts recorded under non denaturing conditions of (A) NarJT and (B) NarJT in the presence of the NarG(1–15) peptide. Each panel (A and B) is composed of the ESI-MS spectra (top) and the corresponding drift chart represented in drift time versus m/z (bottom) obtained by Ion Mobility. Ratios intensities according to the TIC of each spectrum are given in drift charts. Curves depicted in black correspond to drift time curves identified for each NarJT conformers. In panel A, respective charge states of NarJT conformers (Conformer A = filled circle circled, Conformer B = filled circles, Conformer C = diamond) are labeled both on mass spectrum and mobility chart. In panel B, open circles correspond to NarJT alone (conformer B*); filled circles correspond to NarJT complexed with its partner peptide (conformer B*P); filled squares correspond to NarJT complexed with 2 molecules of its partner peptide (conformer B*2P). Arrows in panel B denote +1 and +2 CS of NarG(1–15) peptide observed all along the TWIM cell. A drift time value of 1 bin corresponds to 90 µs.

To gain further insights into the structural flexibility observed through a large distribution of inter-label distances, mass spectrometry experiments performed under non denaturing conditions with or without ion mobility segregation were carried out on NarJT alone (Figure 5A) and in presence of the NarG(1–15) peptide (Figure 5B). Interestingly the ESI-MS experiment revealed a tri-modal Charge State Distribution (CSD) suggesting that NarJT exists under at least three conformers: namely conformer A corresponding to +12 to +21 charge states (CS), conformer B corresponding to +9 to +11 CSs and conformer C corresponding to +6 to +8 CSs (Figure 5A). Relative to the total ion current, conformers A, B and C account for 25%, 73% and 2%, respectively. Calculation of the average charge state (Z_av_) (Table 2) allows to propose a compactness scale based on protonation propensity [63], with conformer A being the most open form and conformer C the most compact one. Indeed, folded proteins with compact structures give rise to gaseous ions carrying a relatively low number of charges and presenting a narrow CSD centered on high *m/z* values. Final proof of the existence of different conformers in equilibrium was provided by IM experiments. As emphasized in Figure 5A NarJT drift chart allowed distinguishing 3 different drift time curves supporting the presence of 3 conformers in equilibrium in agreement with distinct CSDs.

**Table 2:** ESI-MS and IM data analysis depicting conformational species of NarJT either alone or complexed with NarG(1–15) peptide.

Sample	Z_av_	Ratios in %	CSD	CCS_av_ in Å^2^
NarJT conformer A	16.09	25	+12–+21	N.C.
NarJT conformer B	9.97	73	+9–+11	2537
NarJT conformer C	7.14	2	+6–+8	N.C.
NarJT (B*)	8.65	44	+7–+10	2600
NarJT (B*P)	9.68	54	+7–+11	2453
NarJT (B*2P)	8.75	2	+8–+11	N.C.

Average charge states (Z_av_), ratios intensities in % relative to TIC (indicated values were given with a ±0.5% standard error), charge state distribution (CSD) and averaged collision cross section (CCS_av_) values in Å^2^ calculated for +8 to +10 CS of NarJT conformers detected when alone or in mixture with the NarG(1–15) peptide. B*2P stands for NarJT in complex with two peptides.

To address the impact of NarG(1–15) peptide binding on NarJT conformational ensemble, mass spectrum was recorded under the same experimental conditions (Figure 5B). In addition to the +2 and +1 CS of the free NarG(1–15) peptide, NarJT alone or complexed to one or two peptide molecules were observed. Experimental masses of these respective species were in agreement with predicted ones (Table S2). Relative to the total ion current, ratios of the different NarJT species were 44%, 54% and 2%, for NarJT alone, NarJT complexed to its peptide and NarJT complexed with two peptides, respectively. The very weak abundance of the NarJT protein complexed with two peptides and the concomitant increase of this species using a higher peptide protein ratio suggest a non-specific binding of the second peptide molecule. Interestingly, the tri-modal CSD observed for NarJT alone (Figure 5A) was replaced herein by two new CSDs having close patterns and corresponding to NarJT alone (CSD = +7 to +10 CSs) and NarJT complexed to its partner peptide (CSD = +7 to +11 CSs), respectively (Figure 5B). Noteworthy is the fact that CSDs and drift time curves of these two new components were close to the major conformer B observed for NarJT alone and as a consequence were renamed B* (NarJT free of peptide) and B*P (NarJT in complex with one peptide) (Figure 5). Origin of the B* conformer was given considering that, in previous work, NarJT-peptide binding was shown to be mostly entropy-driven through hydrophobic interactions [18]. Thereof, the complex is most likely partly dissociated upon collision activation in the gas phase which is detrimental for hydrophobic interactions [64] and resulting in nearly equal amount of bound (B*P) and unbound (B*) forms. In addition, it cannot be inferred from close CSDs values between B*, B*P and B conformers that they all have the same conformation.

To better document the conformational changes occurring when NarG(1–15) peptide was added to NarJT, the Z_av_ was calculated. It appears that both B* and B*P undergo a decrease in the Z_av_ values as compared to the conformer B, indicating a reduction of the accessible protein surface for protonation (Table 2). In this context the additional charge observed for B*P was assumed to be carried out by NarG(1–15) peptide. Finally, averaged collision cross-section (CCS_av_) values from the +8 to +10 CS of B, B* and B*P were calculated (Table 2). While the CCS_av_ of B* increased as compared to B*P due to complex dissociation in the gas phase, a small decrease of the CCS_av_ occurred as the result of complex formation when comparing B to B*P. Altogether, these results suggest that binding of the NarG(1–15) peptide led to the selection of conformer B followed by subtle structural changes.

Overall, our ESI-MS and ESI-TWIM-MS results provide additional support to the structural dynamics of NarJT and suggest that complex formation arise from the conformational selection of one of the NarJT conformers *ie* conformer B.

## Discussion

An important role for protein flexibility has been widely discussed in the literature [1], [2], [3], [65] and represents a technical challenge when describing protein interactions. Previous NMR studies conducted on *E. coli* NarJ both in absence and in presence of the NarG(1–15) peptide showed the absence of a number of peaks in the ^1^H,^15^N-HSQC spectrum which precluded complete assignment of the residues [18]. Moreover, global change of the ^1^H,^15^N-HSQC spectrum of NarJ upon peptide binding has been interpreted as the result of a conformational change upon complex formation without inferring the underlying molecular mechanisms. Altogether, these NMR studies conducted for the first time on a dedicated chaperone of the NarJ family did not provide a detailed picture of the conformational dynamics. Herein the combination of site-directed spin labeling EPR spectroscopy and IM mass spectrometry has provided a unique window on the conformational substates of the dedicated chaperone NarJ and during the partner binding process, revealing distinct molecular species and conformational dynamics. More importantly, our study explains how structural flexibility of the dedicated chaperone can be exploited for binding of the N-terminus of the metalloprotein during the folding and assembly process.

Strong evidences for the existence of a structural flexibility came from two complementary approaches revealing that the NarJT protein was represented by at least three discrete conformations. DEER experiments performed on a doubly-labeled NarJT variant in frozen state show a broad distance distribution with multiple maxima (Figure 3B and Figure 4B) while IM experiments allow distinguishing three conformers (Figure 5A). Importantly, such structural equilibrium was shown to disappear to the profit of a distance distribution centered on one single maximum or a single protein conformation when the NarG(1–15) peptide was added. It is worth to notice that, by the use of singly spin-labeled proteins, the E119 residue present in a funnel-shaped hydrophobic cavity conserved in all members of the dedicated chaperone is identified as being part of the binding site for the N-terminus of NarG. In particular, the singular location of the E119 residue within this cavity makes it a valuable tool to sense NarG(1–15) peptide binding. Simulation of the EPR spectra revealed two components of equal weights for the E119C variant (Figure 2) reflecting two different environments of the spin probe. Remarkably, the drastic change in the EPR spectrum observed upon complex formation revealed the conversion of these two populations into a major one of more restricted conformational space resulting from the presence of the NarG(1–15) peptide. These observations are reminiscent of the redistribution of the conformational substates of NarJT obtained upon complex formation from IM experiments.

From a mechanistic point of view, it is important to ask when the conformational transition occurs during the binding process. Several models have been proposed to explain the conformational changes observed between the bound and unbound forms of proteins. In the induced fit mechanism the ligand induces a conformational change upon binding while in the preexisting equilibrium model protein samples an ensemble of conformations at equilibrium conditions and a fraction of which is predisposed to recognize and bind selectively a particular ligand.

In our study, two lines of evidence are highly suggestive of a conformational selection mechanism operating during binding of the N-terminus of NarG by the NarJ chaperone. At first, the distance distribution with one single maximum revealed by DEER in the bound form of NarJT was part of the large and composite distance distribution obtained in the unbound form (Figures 3 and 4). Secondly, the new NarJT conformations (B* and B*P) observed by IM after peptide addition are highly similar to the major conformer B observed for NarJT alone since CSDs and drift time curves of B* and B*P conformers were close to B (Figure 5) suggesting that B* conformer would derive from B. Further analysis revealed a decrease of Z_av_ value associated with B* and B*P that is related to a reduced surface accessibility for protonation as compared to B (Table 2). It is noticeable that B*P Z_av_ value was found to carry one more charge in average as compared to B*. We hypothesized that the presence of an additional charge on B*P could be linked to the binding of the NarG(1–15) peptide forming an amphiphilic α-helix [18], [19] with the charge retained specifically on the outward polar α-helix face. Second CCS_av_ values that reflect global gas phase conformation were compared to gain better insights upon global conformational changes occurring both when partner peptide binds to B (*ie* B*P) or dissociate from B*P (ie B*). The observation of a decrease in CCS_av_ value when the peptide binds to B conformer was consistent with previous calorimetric data showing that binding induces conformational changes leading to a more compact structure as deduced from higher excess partial molar heat capacity of the complex [18]. Interestingly, dissociation of the complex in the gas phase gave rise to a higher CCS_av_ value for the unbound B* form which denote a more open structure.

One tentative model that is consistent with our experimental observations is that NarJT protein is represented by at least 3 conformers in equilibrium and that peptide binding results from the selection of an accessible conformation and its further rearrangement induced upon peptide recognition. An additional interesting observation is that the entropy of binding (Δ*S*_bind_) obtainable by calorimetric methods is composed of contributions associated with the protein, the ligand and the solvent. Zakian et al showed a large and positive Δ*S*_bind_, which accounts for most of the binding process in the NarJT-NarG(1–15) peptide complex [18]. If such positive entropy variation was originally interpreted as the result of hydrophobic contacts or the loss of water-mediated hydrogen bonds, our results likely support the additional contribution of conformational entropy which has been considered important for molecular recognition [66]. At this stage, it is worth mentioning the absence of structural information to date concerning the mode of interaction between the dedicated chaperones and the N-terminus of their cognate metalloprotein partner, in particular no X-ray structure of such a complex is available so far. During the last decade, many debates have concerned the exact location of the N-terminus binding site within the dedicated chaperone. By revealing structural dynamic at play during the recognition and binding process, our results provide herein a framework for understanding the mode of interaction.

Several studies reported that proteins have often evolved to bind multiple targets, including proteins, peptides, DNA, and small molecule substrates. The simplest way to achieve multispecificity is to recognize each target through a distinct binding interface and multiple binding domains. However, X-ray structures of several dedicated chaperones revealed the existence of single-domain proteins sharing a common all-helical fold with only a low level of sequence identity (Pfam PF02613) [9]. In such cases, conformational flexibility is the most frequent explanation for multispecificity [67], [68]. As demonstrated here for the first time, NarJ samples different conformational substates and we anticipate that this situation may hold true for other members of the family. Structure of those dedicated chaperones may have evolved to allow interaction with a diverse range of partners such as components of metal center biosynthesis machineries, or conversely, binding partners may have taken advantage of preexisting conformational diversity [3], [69]. Both processes may be important in the evolution of molecular recognition.

Furthermore no information is available concerning the binding interface for the multiple partners of NarJ involved in metal center delivery during the assembly process of the cognate metalloprotein. In this context, stabilization of a specific conformer of NarJ with NarG(1–15) peptide binding and thus redistribution of the protein conformational ensembles recalls allostery, a mechanism by which binding of the ligand at one site can affect binding of others through a propagated change in the protein shape [70], [71]. Interestingly, Volkman et al showed that the inactive response regulator NtrC, a single-domain protein, samples the active state conformation even in the absence of the ligand [72]. Allostery thus derives from a redistribution of the conformational ensemble [70]. Overall, the shift in population resulting from peptide binding could be one of the keys to facilitate subsequent binding of additional partners at yet unidentified sites of NarJ. Such a situation may also hold true in other metalloproteins of interest such as hydrogenases or ureases which rely on dedicated chaperones for their folding and function [73], [74].

## Supporting Information

Figure S1 CD spectra of NarJ, NarJT and all cysteine variants of NarJT before (A) and after (B) spin labeling.
(TIFF)

Table S1 NarG(1–15) peptide binding activity as estimated by intrinsic tryptophan fluorescence quenching.
(PDF)

Table S2 Calculated, theoretical molecular weights and mass errors given in Da and ppm of NarJT species detected under non denaturing conditions.
(PDF)
