# Supplementary figures and images for: Conformational Selection Underlies Recognition of a Molybdoenzyme by Its Dedicated Chaperone
Source: PLoS One. 2012 Nov 19;7(11):e49523. doi: 10.1371/journal.pone.0049523 (PMC3501500; doi:10.1371/journal.pone.0049523)

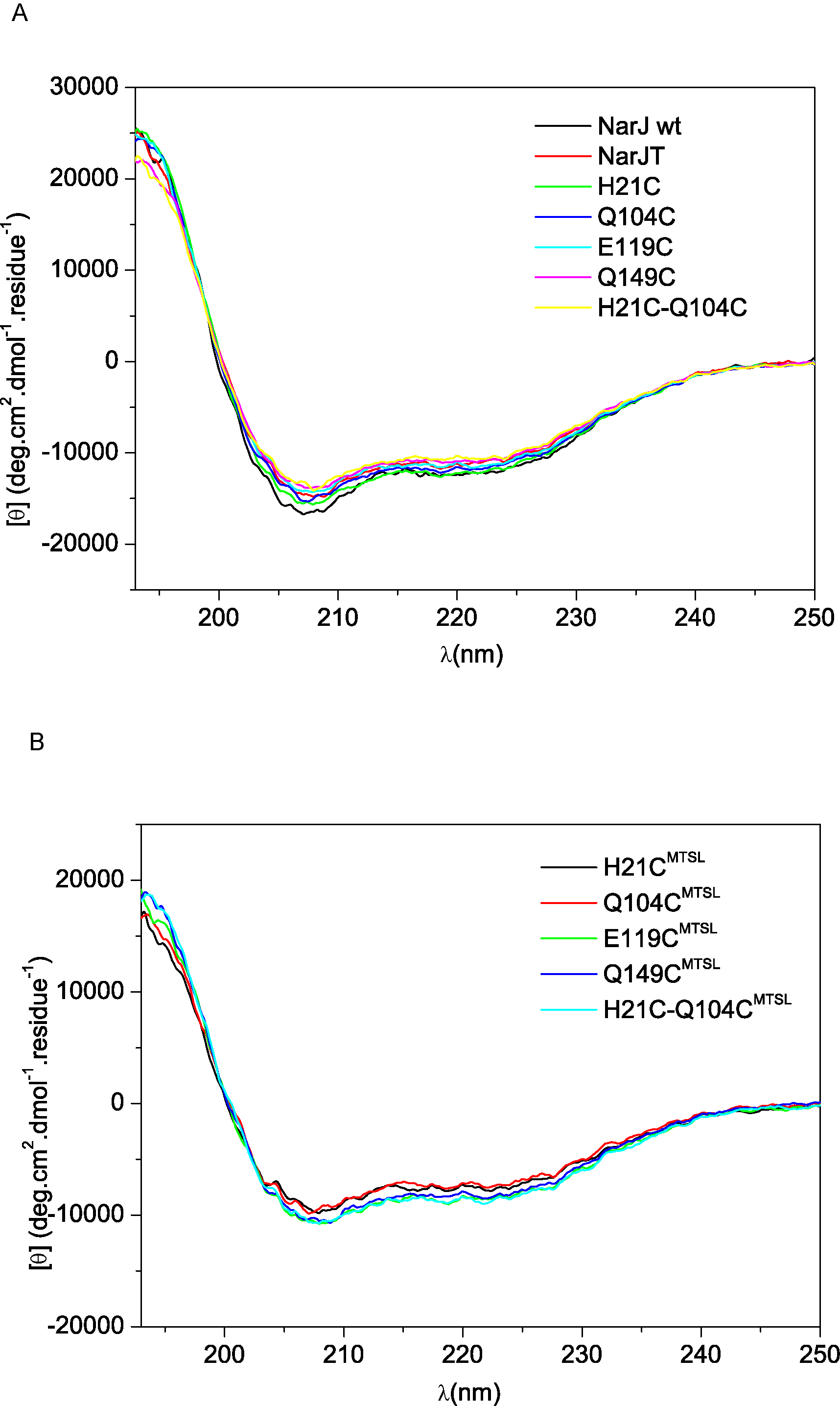

Supplement: Figure S1 — CD spectra of NarJ, NarJT and all cysteine variants of NarJT before (A) and after (B) spin labeling. (TIFF) [file pone.0049523.s001.tiff]
