# Supplementary material for: Conformational Selection Underlies Recognition of a Molybdoenzyme by Its Dedicated Chaperone
Source: PLoS One. 2012 Nov 19;7(11):e49523. doi: 10.1371/journal.pone.0049523 (PMC3501500; doi:10.1371/journal.pone.0049523)
Supplement: Table S1 — NarG(1–15) peptide binding activity as estimated by intrinsic tryptophan fluorescence quenching. (PDF) [file pone.0049523.s002.pdf]

| **Sample** | ***K*_D_ (µM)** |
| --- | --- |
| Unlabeled protein |  |
| NarJT | 0.55 ± 0.11 |
| NarJT H21C | 0.78 ± 0.15 |
| NarJT Q104C | 0.30 ± 0.10 |
| NarJT E119C | 0.54 ± 0.11 |
| NarJT Q149C | 0.35 ± 0.10 |
| NarJT H21C,Q104C | 0.26 ± 0.10 |
| MTSL-labeled protein |  |
| NarJT H21C | 0.45 ± 0.10 |
| NarJT Q104C | 0.35 ± 0.10 |
| NarJT E119C | 0.32 ± 0.10 |
| NarJT Q149C | 0.28 ± 0.07 |
| NarJT H21C,Q104C | 0.24 ± 0.06 |

Indicated values are the average of at least three independent measurements.
