# Supplementary material for: Conformational Selection Underlies Recognition of a Molybdoenzyme by Its Dedicated Chaperone
Source: PLoS One. 2012 Nov 19;7(11):e49523. doi: 10.1371/journal.pone.0049523 (PMC3501500; doi:10.1371/journal.pone.0049523)
Supplement: Table S2 — Calculated, theoretical molecular weights and mass errors given in Da and ppm of NarJT species detected under non denaturing conditions. (PDF) [file pone.0049523.s003.pdf]

| **Sample** | **Expt^al^ MW**  **(Da)** | **Theo^al^ MW**  **(Da)** | **Mass error (Da)** | **Mass error (ppm)** |
| --- | --- | --- | --- | --- |
| NarJT conformer B | 22000.3 ± 0.5 | 21999.93 | 0.37 | 17 |
| NarJT (B*) | 22001.2 ± 0.5 | 21999.93 | 1.27 | 58 |
| NarJT (B*P) | 23951.8 ± 0.5 | 23951.23 | 0.57 | 25 |
| NarJT (B*2P) | 25904.5 ± 0.5 | 25902.5 | 2.00 | 77 |
